# Dysregulation of Vascular Endothelial Progenitor Cells Lung-Homing in Subjects with COPD

**DOI:** 10.1155/2016/1472823

**Published:** 2016-05-12

**Authors:** Brittany M. Salter, Fizza Manzoor, Suzanne Beaudin, Melanie Kjarsgaard, Parameswaran Nair, Gail M. Gauvreau, Roma Sehmi

**Affiliations:** ^1^Department of Medicine, McMaster University, 1280 Main Street West, Hamilton, ON, Canada L8N 3Z5; ^2^Firestone Institute for Respiratory Health, Asthma Research Group, St. Joseph's Healthcare, 50 Charlton Avenue East, Hamilton, ON, Canada L8N 4A6

## Abstract

Chronic obstructive pulmonary disease (COPD) is characterized by fixed airflow limitation and progressive decline of lung function and punctuated by occasional exacerbations. The disease pathogenesis may involve activation of the bone marrow stimulating mobilization and lung-homing of progenitor cells. We investigated the hypothesis that lower circulating numbers of vascular endothelial progenitor cells (VEPCs) are a consequence of increased lung-sequestration in COPD. Nonatopic, current or ex-smokers with diagnosed COPD and nonatopic, nonsmoking normal controls were enrolled. Blood and induced sputum extracted primitive hemopoietic progenitors (HPCs) and VEPC were enumerated by flow cytometry. Migration and adhesive responses to fibronectin were assessed. In sputum, VEPC numbers were significantly greater in COPD compared to normal controls. In blood, VEPCs were significantly lower in COPD versus normal controls. There were no differences in HPC levels between the two groups in either compartment. Functionally, there was a greater migrational responsiveness of progenitors from COPD subjects to stromal cell-derived factor-1alpha (SDF-1*α*) compared to normal controls. This was associated with greater numbers of CXCR4^+^ progenitors in sputum from COPD. Increased migrational responsiveness of progenitor cells may promote lung-homing of VEPC in COPD which may disrupt maintenance and repair of the airways and contribute to COPD disease pathogenesis.

## 1. Introduction 

Chronic obstructive pulmonary disease (COPD) is characterized by fixed airflow limitation and progressive decline of lung function punctuated by frequent exacerbations [1, 2]. Pulmonary vascular endothelial dysfunction, as shown by alterations in vessel structure, dysfunctional endothelial cellular growth, and resistance to apoptosis, is a characteristic finding of COPD [3, 4]. Endothelial cell damage and abnormal function of pulmonary vessels triggers pulmonary vascular remodeling, leading to pulmonary hypertension [5]. The mechanism behind endothelial repair and regeneration in COPD is not well understood. Evidence suggests that COPD is associated with a systemic process involving activation of the bone marrow stimulating increased turnover and trafficking of vascular endothelial progenitor cells (VEPCs) that engraft to the lung. Once within the pulmonary tissue, VEPC can differentiate into endothelial cells, contributing to tissue repair and maintenance, governed by locally elaborated growth factors [6].

Studies have shown that circulating levels of VEPC in COPD are lower at baseline compared to normal subjects [7], increase during exacerbations [8], and correlate with hypoxemia, airway obstruction [9], and disease severity [10]. This suggests that a reduction in circulating VEPC levels may be due to a defective bone marrow response in COPD. An alternative, as yet uninvestigated, interpretation may be that diminished numbers in the peripheral blood are caused by increased sequestration of VEPC in the airways promoting pulmonary vascular remodeling and neovascularization.

Both vascular endothelial growth factor (VEGF) and hepatocyte growth factor (HGF) are proangiogenic factors that have different action profiles [11, 12]. VEGF is a marker of activation of the hypoxic pathway [13], whereas HGF acts as growth factor and chemoattractant for VEPC [14]. Due to the increased secretion in an injured lung, HGF has an important role in the wound healing of pulmonary epithelium and recruitment of and differentiation of VEPC. This study investigated responsiveness to VEGF and HGF compared to other progenitor chemoattractants such as stromal cell-derived factor-1alpha (SDF-1*α*), in order to understand the factors promoting lung-homing of progenitors in COPD.

We investigated the hypothesis that diminished circulating numbers of vascular endothelial progenitor cells (VEPCs) in subjects with COPD are a consequence of increased lung-sequestration. The novel findings of this study are that reduced number of circulating VEPCs but not HPCs in COPD is associated with increased sputum levels. We found that progenitors from COPD subjects had a greater migrational response than normal subjects, suggesting that increased homing of VEPC from the blood to the airways may be an important component to the pathogenesis of COPD.

## 2. Methods 

### 2.1. Subjects

COPD (*n* = 9) subjects (18–75 years of age) were nonatopic, current or ex-smokers (at least 15 pack year smoking history), with chronic bronchitis, a FEV_1_ < 70% predicted and prebronchodilator FEV_1_/FVC < 0.70, and a postbronchodilator (400 *μ*g of salbutamol) of FEV_1_/FVC < 0.70. Subjects were clinically stable at the time of the study without exacerbations or oral steroid treatment for 4 months prior to study. As shown in Table 1, most COPD subjects (66.67%) were on regular bronchodilator treatment. Eight out of 9 COPD subjects had no cardiovascular and metabolic diseases. Normal controls (*n* = 8) (18–75 years of age) were nonatopic, nonsmokers, with FEV_1_/FVC > 0.70. The study protocol was approved by Hamilton Integrated Research Ethics Board (#07-2914), and all subjects provided written informed consent.

### 2.2. Study Design

Subjects attended the clinic for medical history, skin-prick testing to assess allergy to common aeroallergen extracts, spirometry before and after salbutamol to assess FEV_1_ and vital capacity, sputum induction, and venous blood collection (120 mL).

### 2.3. Sputum Induction

Sputum (SP) samples were induced using 0.9% normal saline and mucous plugs were selected and dispersed with dithiothreitol, as previously described [15]. Cytospins were prepared from sputum cells and stained with Diff-Quick for differential cell counts.

### 2.4. Immunofluorescence Staining and Flow Cytometric Analyses

Enumeration and phenotypic analyses of peripheral blood (PB) and SP HPC were assessed by flow cytometry using monoclonal antibodies (mAbs) to lineage markers: CD45-FITC, CD34-PE, and CD133, IL-5R*α*, IL-3R*α*, or GMCSFR*α*, chemoattractant marker CXCR4, and adhesion markers *β*1 integrins VLA4 (CD49d) and VLA5 (CD49e) and *β*2 integrin Mac-1 (CD11b) which were conjugated to PerCP. PB and SP extracted cells were suspended in FACS buffer, immunostained with isotype or specific mAbs (4°C, 30 minutes), then washed with NaN_3_, and fixed in PBS plus 1% paraformaldehyde. Cells were acquired using a Becton Dickinson FACScan flow cytometer equipped with 3-colour argon ion laser. Analyses of FACS data were performed using a four-step gating strategy as previously described [16]. HPCs were identified as FSC^low^/SSC^low^/CD45^dim^/CD34^high^ events and VEPCs were identified as FSC^low^/SSC^low^/CD45^dim^/CD34^high^/CD133^high^ events.

### 2.5. Progenitor Cell Isolation

Blood was drawn into heparin (1000 units/mL) and layered onto 60% Percoll, for density gradient fractionation to isolate low-density mononuclear cells (MNC). The interface cells were washed with McCoy's 5A and subject to adherence on plastic to deplete macrophages. Nonadherent MNC fraction was subjected to positive selection of CD34^+^ cells using magnetic beads as previously described [17].

### 2.6. Migrational Responsiveness

Migrational responsiveness of PB CD34^+^ cells was assessed by intracellular filamentous (F) compared to globular (G) actin ratio [18]. Briefly, enriched CD34^+^ cells were washed and resuspended in IMDM with 0.25% BSA (1 × 10^6^ cells/mL) and prewarmed for 10 minutes at 37°C, followed by coincubation with SDF-1*α* (10 ng/mL), HGF (50 ng/mL), VEGF (50 ng/mL), or diluent at various time-points (0, 30, 60, 120, 300, and 600 seconds). Cells were fixed for 15 minutes, washed, and then permeabilized for 5 minutes. This was followed by incubation for 20 minutes with 1 U/mL of Alexa Phalloidin (detecting F-actin) and Texas Red DNAse I (detecting G-actin), resuspended in PBS and acquired FACScan flow cytometer. F : G ratios were then calculated for each cell sample as previously described [18].

### 2.7. Adhesion Assay

Adhesion assays were performed as previously described [19]. Briefly, enriched PB-derived CD34^+^ cells were incubated in fibronectin-coated plates for 45 minutes at 37°C (27,500 cells/well) and exposed to SDF-1*α* (1, 10, and 100 ng/mL), HGF (1, 50, and 100 ng/mL), VEGF (1, 50, and 100 ng/mL), or diluent for 30 minutes at 37°C. Adherent cells were then recovered with cell dissociation buffer and Iscove's modified Dulbecco's medium plus 10% FBS and then stained with antibodies (CD45 and CD34 mAb) for flow cytometry enumeration and analysis as previously described [19].

### 2.8. Statistical Analyses

Between groups analyses were performed using nonparametric tests (Mann-Whitney *U* test) and ANOVA was used within group analyses. Significance was set to a *p* value of <0.05.

## 3. Results

### 3.1. FACS Enumeration of Progenitor Cells in Blood and Sputum

**Figure 1:**
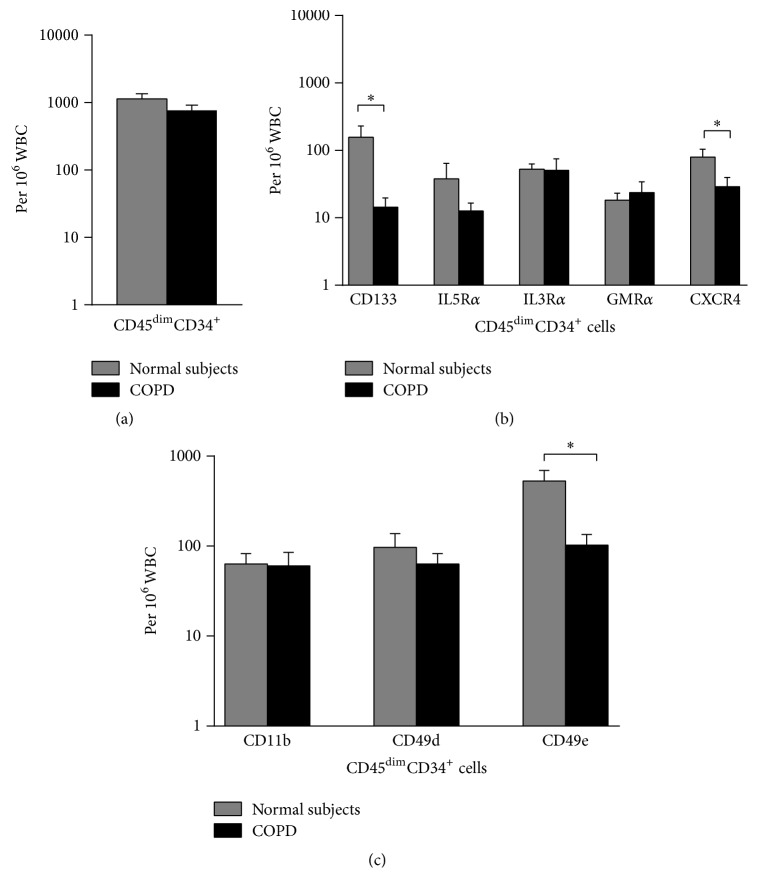
(a) Enumeration, (b) phenotyping, and (c) assessment of adhesion receptors by flow cytometry on blood HPC progenitor cells collected from COPD patients (*n* = 9) and normal nonatopic controls (*n* = 8). Data are presented as mean ± SEM; ^*∗*^ *p* < 0.05 between group comparisons.

Enumeration by sequential multigating multiparametric analyses of PB samples showed nonsignificant difference in the absolute number of HPCs (CD45^dim^CD34^+^ cells) in COPD subjects versus normal subjects, (765 ± 151 and 1131 ± 227/10^6^ WBC, resp.) (Figure 1(a)). Further phenotypic analyses of the progenitor cell population showed significantly lower numbers of VEPCs (CD45^dim^CD34^+^CD133^+^ cells) in PB from COPD versus normal subjects (14 ± 5 and 157 ± 74/10^6^ WBC, *p* < 0.05) (Figure 1(b)). The absolute number of PB HPCs expressing CXCR4 was significantly lower in subjects with COPD compared to normal controls (29 ± 11 and 89 ± 24/10^6^ WBC, *p* < 0.05) (Figure 1(b)).

There were no significant differences in the expression of the lineage-commitment markers between the two groups as shown in Figure 1(b). In contrast there were significantly greater absolute numbers of PB HPCs expressing adhesion markers, CD49e in normal subjects compared to COPD subjects (529 ± 165 and 102 ± 32 cells/10^6^ WBC, *p* < 0.05) (Figure 1(c)). In contrast, there were no significant differences in the expression of CD11b or CD49d between the two groups (Figure 1(c)).

**Figure 2:**
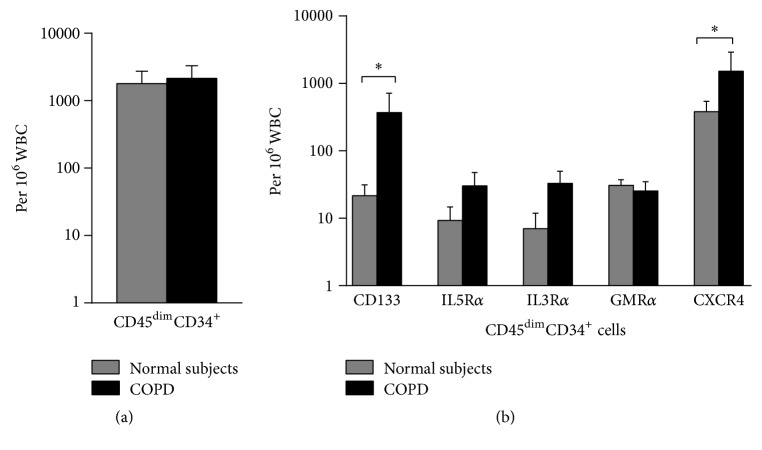
(a) Enumeration and (b) phenotyping by flow cytometry of sputum progenitor cells from COPD patients (*n* = 9) and normal nonatopic controls (*n* = 8). Data are presented as mean ± SEM; ^*∗*^ *p* < 0.05 between group comparisons.

In SP, there was no significant difference in differential cell counts between the subject groups (Table 2). In addition, there was a no difference in the absolute number of HPCs, between COPD and normal subjects (2154 ± 108 and 1805 ± 93 cells/10^6^ WBC, resp.) (Figure 2(a)). In contrast, the absolute number of SP VEPCs was greater in COPD compared to normal subjects (370 ± 346 and 22 ± 10 cells/10^6^ WBC, *p* = 0.038) (Figure 2(b)). Similarly, the absolute number of SP HPCs expressing CXCR4 was increased in COPD compared to normal subjects (1521 ± 14 and 343 ± 120 cells/10^6^ WBC, *p* < 0.05) (Figure 2(b)). There were no correlations found between the absolute numbers of VEPCs in PB or SP and lung function measurements, likely due to the small sample size (data not shown). Due to low number of absolute cells recovered from induced sputum samples, additional analyses of adhesion molecules could not be performed in the same samples.

### 3.2. Migrational Responsiveness of Blood CD34^+^ Cells: F/G Actin Assay

**Figure 3:**
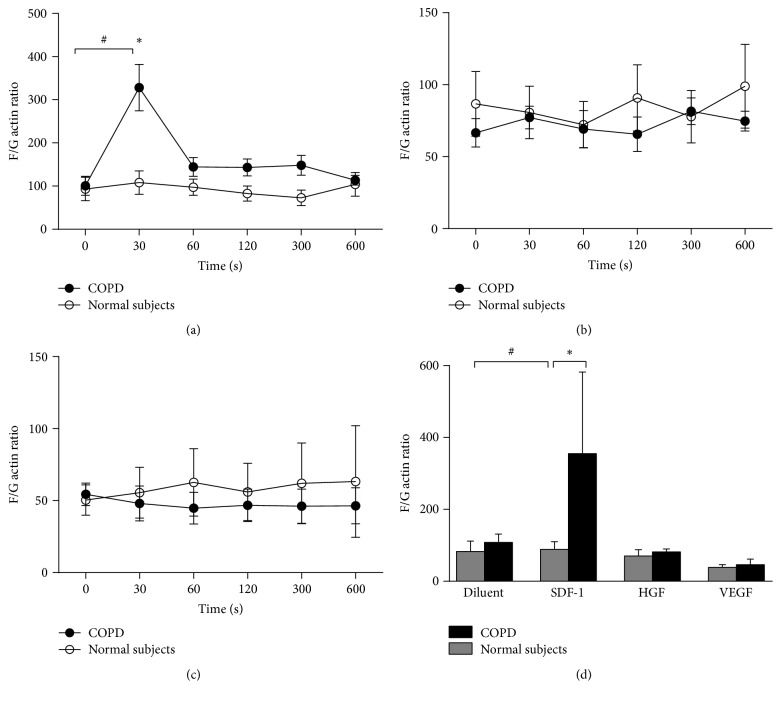
Migrational responsiveness to blood HPC assessed by intracellular F/G actin ratio following incubation with predetermined optimal dose of (a) SDF-1*α* (10 ng/mL), (b) HGF (50 ng/mL), or (c) VEGF (50 ng/mL) at varying time-points. Summary F/G actin ratio data are presented in (d) following 30 seconds of stimulation. Data are presented as mean ± SEM (*n* = 8); ^*∗*^ *p* < 0.05 between group comparisons and ^#^ *p* < 0.05 within group comparisons to diluent control.

A phalloidin intracellular staining was performed to enumerate the formation of F-actin compared G-actin. This is considered a surrogate assay where the level of formation of F-actin is associated with cellular migrational response. Our data show that PB HPC isolated from COPD subjects had significantly greater migrational responsiveness to SDF-1*α* compared with HPC from normal subjects (Figure 3). No significant migrational response was elicited by HGF or VEGF in either COPD or normal subjects at any time-point (Figure 3). All concentrations of the chemoattractants were previously optimized in pilot experiments.

### 3.3. Adhesive Responses of Progenitor Cells

**Figure 4:**
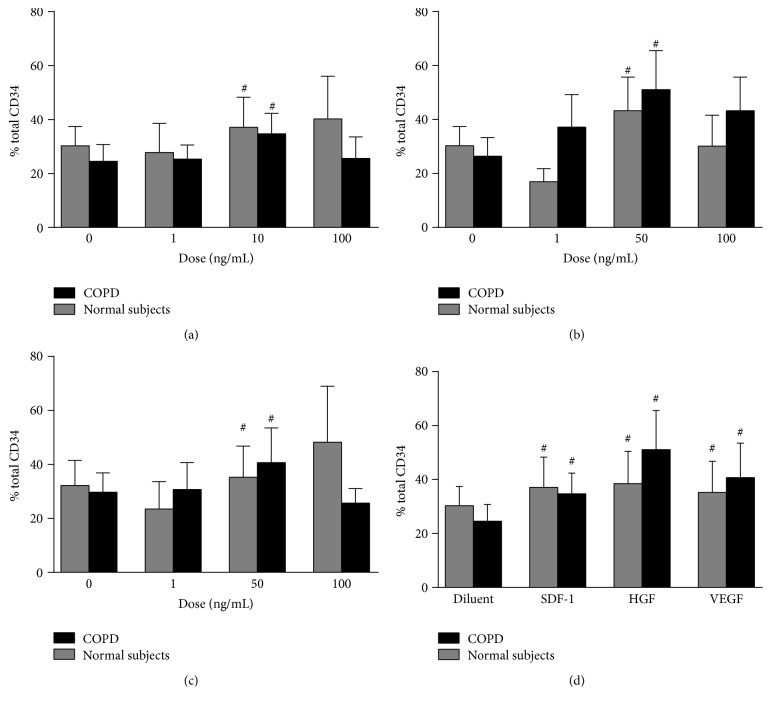
Enumeration of adhesion to fibronectin of blood HPC from COPD and normal subjects to (a) SDF-1*α*, (b) HGF, and (c) VEGF. (d) Comparison of the optimal adhesive response to SDF-1*α* (10 ng/mL), HGF, and VEGF (both 50 ng/mL) shows no significant difference between the two groups of subjects. Data are presented as mean ± SEM (*n* = 6); ^#^ *p* < 0.05 within group comparisons to diluent control.

The transcellular migrational capacity of cells is a combination of the adhesive properties of the cells and the migratory capacity. We investigated the ability of peripheral blood HPC to adhere to extracellular components such as fibronectin. Our data showed that there were increased adhesive responses of HPC to fibronectin when the cells were stimulated with a wide concentration range of SDF-1*α*, HGF, or VEGF (Figures 4(a), 4(b), and 4(c)). However, there was no significant difference between the two groups although there was a trend for greater responsiveness of HPC from COPD compared with normal subjects toward HGF (50 ng/mL) and VEGF (50 ng/mL) stimulation (Figure 4(d)). This was not seen with SDF-1*α* (10 ng/mL) (Figure 4(d)).

## 4. Discussion

COPD is characterized by structural and functional changes in the pulmonary parenchyma, central and peripheral respiratory tract, and pulmonary circulation. Evidence suggests that HPCs and VEPCs play important roles in angiogenesis, working in concert to maintain and repair the capillary endothelium. HPCs migrate to areas of injury, where they produce growth factors that induce blood vessel sprouting and endothelial proliferation [20]. VEPCs produce growth factors stimulating local angiogenesis and incorporate directly into existing microvessels to act as building blocks to form new vasculature [21]. VEPCs have been shown to have therapeutic value, acting as repair cells, in coronary artery diseases, which are incorporated into the endothelium following ischemia [22–24] and after endothelial injury [25, 26], thereby maintaining tissue vasculature. Reduced circulating VEPC numbers and function, as well as pulmonary capillary density shown in subjects with chronic disease [27–35], and failure to maintain the capillary endothelium may be an underlying cause of chronic respiratory diseases [36, 37].

VEGF is a potent chemoattractant and survival factor for both HPC and VEPC, mediated through VEGF receptors [38, 39]. Mouse models have shown that blocking of VEGF receptor signalling leads to alveolar capillary dropout with secondary death of alveolar epithelial cells, leading to emphysema [40]. Human studies have shown that in COPD and emphysema there are decreased circulating VEPC and bronchiolar expression of VEGF, as well as increased cell death [7–9, 41–43]. The lack of mobilization of progenitors from the bone marrow to the periphery could be in part due to dysfunctional interaction between VEGF and the VEGF receptor on progenitor cells. It is postulated that impaired progenitor cell mobilization, function, and lung-homing may contribute to failed repair and maintenance of pulmonary vasculature. This is consistent with the findings of Liu and Xie who reported that migration of VEPC and expression of CXCR4 were reduced in COPD [44]. Here we show for the first time that COPD subjects have increased absolute numbers of VEPCs, as well as HPC expressing CXCR4 in the airways.

In transmigration assays, blood-derived HPC from subjects with COPD had greater migrational responsiveness to SDF1-*α* compared to HPC from normal subjects. These data support our hypothesis that decreased numbers of circulating HPCs in COPD may be due to increased lung-homing. This is in part supported by reports of increased numbers of CD45^+^CD133^+^ cells on endothelial surfaces and intimal layers of the pulmonary arteries in smokers with COPD [45]. In contrast, we found no differences in adhesion to fibronectin between the two groups, suggesting that only certain aspects of total progenitor cell population transmigration are affected in COPD.

Notably, although we found marked differences in VEPCs between COPD and normal subjects, we found no difference in total absolute numbers of HPCs. This is consistent with Janssen et al. who found no difference in circulating CD45^dim^CD34^+^ cells between COPD and healthy controls [41]. We conclude that although HPC absolute numbers within the circulation and airways did not change between the two groups, subtypes of HPC did in fact change, indicating that a specific phenotype of HPC is important in COPD. This is supported by finding no difference between the two groups with respect to HPC numbers expressing lineage markers IL-3R*α*, IL-5R*α*, and GM-CSFR*α*. These receptors can enumerate HPC that have the potential to differentiate into eosinophil/basophil-CFU or granulocyte/macrophage-CFU, which have been shown to contribute to the pathogenesis of asthma [46, 47]. However, it is possible that these subtypes do not play an important role in COPD, as opposed to VEPC.

Notably, we found no difference in the migrational response of HPC to HGF or VEGF between the two groups. It is interesting in cells from COPD subjects there was no increased responsiveness to VEGF, given that this is a potent chemoattractant, yet we saw an increase in VEPC within the airways. Kim et al. demonstrated that, in COPD, circulating VEPC showed significantly lower migratory capacity at baseline and in response to VEGF than those from normal nonsmoking controls [48]. However, it is now accepted that EPC-CFU are derived from cells of monocyte origin, and although EPC-CFU express endothelial markers and provide an accurate measure of the activity of circulating mononuclear cells to form endothelial cells, these colonies are unrelated to circulating CD34^+^ or CD133^+^ progenitor cells [49]. We conclude that differences between these studies may be attributed to the use of HPC versus EPC-CFU, as well as different methodological techniques to measure migration.

We were able to demonstrate that, in the presence of optimal concentrations of SDF-1*α*, HGF, and VEGF, there was significant adhesion to fibronectin, compared to the diluent. However, there was no difference in the magnitude of the adhesive response between the two groups. We have previously shown that adhesion of progenitors to fibronectin is mediated by either VLA4 or VLA5 to the same extent with no additive effect [19]. This may explain the findings of significant differences in levels of CD49e between the two groups, yet the levels of CD49d were comparable, thus maintaining a similar adhesive responsiveness to fibronectin.

This study had some notable limitations. Firstly, 8 of 9 of the COPD subject had no cardiovascular risk factors aside from smoking or metabolic diseases. Although this is not representative of the common COPD population, the absence of comorbidities and cardiovascular risk factors allowed us to avoid potential confounding variables on progenitor cell numbers within the circulation and airways [30, 50–54]. Other factors that may impact circulating progenitor cell numbers include gender [55, 56] and age [57]. The percentage of males was 85.7 and 42.9 for COPD subjects and normal subjects, respectively. The mean age of our two groups was statistically significant, with COPD subjects having an average age of 64.2 ± 5.4 and 31.3 ± 4.5 for normal subjects. Indeed, there was no matching for age or gender in the two groups; thus we cannot conclusively attribute changes in VEPC number and migrational responsiveness to COPD alone. Sala et al. showed a statistically significant difference in the age groups; however, they did not find any difference in VEPC numbers between COPD subjects and normal subjects [8]. Our data is consistent with study findings that have both matched for gender and age [7–9, 41]. Moreover, given that age has been shown to reduce progenitor cell numbers, this does not explain the increased VEPC within the airways of COPD patients, suggesting that differences here are independent of age.

Secondly, our study is limited by the lack of healthy nonatopic current or ex-smoker controls. We investigated healthy nonatopic nonsmoker individuals for the control group of this study. The effect of cigarette smoking on progenitor cells is controversial. Some studies have reported an inverse correlation between the numbers of cigarettes smoked and frequency of VEPCs, which increase rapidly following smoking cessation [58, 59]. VEPCs from healthy smokers have reduced proliferative, migratory, and adhesive capacities [60]. Conversely, other studies have reported similar numbers of circulating progenitors in healthy smokers and nonsmokers, suggesting that, in the absence of other cardiovascular risk factors, cigarette smoking does not significantly alter progenitor numbers [7, 41]. Thus, we can reasonably conclude that differences between progenitor cells of COPD subjects and normal subjects is independent of smoking habits and is related to the disease itself.

Lastly, there is no clear consensus exact phenotypic profile for identifying VEPC. Palange and Liu et al. identified EPCs as cells positive for CD34 and CD133, and, similar to our findings, they found decreased circulating numbers in COPD [9, 44]. Other groups have defined EPCs by the expression of CD34 and CD133, but also the VEGF receptor, KDR [9, 10, 39, 59]. They argued that the use of CD34 and CD133 is not sufficient to distinguish EPCs from other circulating progenitors such as HPCs. Janssen and coworkers utilized a new VEPC definition of CD45^dim^CD34^+^, which further complicates VEPC standardisation [41]. Using our panel developed for three-colour flow cytometry, we enumerated VEPC using a combination of strategies: CD45^dim^CD34^+^CD133^+^. In the future, there is a need to identify more specific phenotypes for VEPCs, to allow for better profiling of distinct cell populations.

We propose two explanations as to the role of progenitor cells in the airways that may contribute to the pathogenesis of COPD. Firstly, upon infiltration to the lungs, the activity of progenitor cells may be dysfunctional, thereby preventing the repair and maintenance of pulmonary vasculature homeostasis, which could in turn promote COPD. Secondly, recruited progenitor cells to the airways may have increased activity, which could lead to elevated inflammation and exacerbated endothelial cell proliferation of the vascular wall, causing pulmonary vessel remodelling and pulmonary hypertension, contributing to the pathogenesis of COPD.

In summary, the findings of this study suggest that in COPD there is increased migrational and adhesive responsiveness of blood-derived progenitor cells and that increased homing to the airways may explain the reduced circulating levels of VEPC and increased numbers in the airways in these subjects. We propose that increased infiltration of VEPC to the airways in COPD may in part contribute to the pathogenesis of this disease although this requires further investigation.

## Figures and Tables

**Table 1:** Subject characteristics of COPD patients and normal subjects.

Characteristic	COPD patients (*n* = 9)	Normal subjects (*n* = 8)
Age, years	64.2 ± 5.4^*∗*^	31.3 ± 4.5
Gender, % male	85.7	42.9
BMI, kg·m^2^	26.7 ± 4.6	27.2 ± 5.2
Smoking status		
Current, *n*%	66.7	0.0
Ex-smoker, *n*%	33.3	16.7
Nonsmoker, *n*%	0.0	83.3
Cigarette pack-years	60.6 ± 22.3^*∗*^	1.3 ± 2.2
Comorbidities		
Hypertension, *n*%	25.0	0.0
Hyperlipidemia, *n*%	25.0	0.0
Statin use, *n*%	16.7	0.0
Pulmonary function		
FEV_1_ Prebronchodilator, % predicted	40.4 ± 20.1^*∗*^	101.8 ± 15.2
FEV_1_ Postbronchodilator, % predicted	47.6 ± 11.5	109.0 ± 14.1
FVC Prebronchodilator, % predicted	56.4 ± 13.8^*∗*^	114.6 ± 15.2
FVC Postbronchodilator, % predicted	0.65 ± 0.1^*∗*^	0.88 ± 0.0
FEV_1_/FVC	0.61 ± 0.07	0.85 ± 0.03

Values are expressed as mean ± SD.

^*∗*^Significant difference (*p* < 0.05) between groups.

**Table 2:** Differential counts of SP samples from COPD patients and normal subjects.

Subject group	Neutrophils (%)	Monocytes (%)	Eosinophils (%)	Lymphocytes (%)	Epithelial cells (%)	Total cells (×10^6^ cells/mL)
COPD (*n* = 9)	35.4 ± 11	44.2 ± 11	3.9 ± 3	0.9 ± 0.1	16 ± 9	3.68 ± 1.1
Normal subjects (*n* = 8)	38.6 ± 5	37.6 ± 7	1.2 ± 0.4	1.3 ± 0.1	21.5 ± 11	2.73 ± 0.95

Data from differential cell counts are expressed as a percent of the total cell count. Values are expressed as mean ± SD. No significant difference was found in any cell type between the two subject groups.
